# Policy recommendations for promoting nuclear medicine therapy in Japan 2025, from the Working Group for promoting nuclear medicine therapy of the Japan Society of Clinical Oncology

**DOI:** 10.1007/s10147-025-02858-3

**Published:** 2025-09-30

**Authors:** Kotaro Suzuki, Hideaki Miyake, Anri Inaki, Shoko Takano, Yasutoshi Kuboki, Takashi Mizowaki, Katsumasa Nakamura, Makoto Ueno, Shigemi Matsumoto, Daisuke Obinata, Tohru Nakagawa, Masato Murakami, Yoshiyuki Majima, Megumu Yokono, Masao Namba, Takayuki Yoshino

**Affiliations:** 1 Division of Urology, Kobe University Graduate School of Medicine, Kobe, Japan; 2 Division of Functional Imaging, Exploratory Oncology Research and Clinical Trial Center, National Cancer Center, Kashiwa, Japan; 3 Department of Nuclear Medicine, Yokohama City University Hospital, Yokohama, Japan; 4 Department of Experimental Therapeutics, National Cancer Center Hospital East, Kashiwa, Japan; 5 Department of Radiation Oncology and Image-Applied Therapy, Graduate School of Medicine, Kyoto University, Kyoto, Japan; 6 Department of Radiation Oncology, Hamamatsu University School of Medicine, Hamamatsu, Japan; 7 Department of Gastroenterology, Kanagawa Cancer Center, Yokohama, Japan; 8 Department of Real World Data R and D, Graduate School of Medicine, Kyoto University, Kyoto, Japan; 9 Department of Urology, Nihon University School of Medicine, Tokyo, Japan; 10 Department of Urology, Teikyo University School of Medicine, Tokyo, Japan; 11 Japan Radiopharmaceuticals Association, Tokyo, Japan; 12 PanCAN Japan, Tokyo, Japan; 13 School of Social Sciences, Waseda University, Tokyo, Japan; 14 Planning Section of Radiopharmaceutical Division, Japan Radioisotope Association, Tokyo, Japan; 15 Department of Global Oncology, National Cancer Center Hospital East, Kashiwa, Japan

**Keywords:** Nuclear medicine therapy, Policy recommendations, ^177^Lu-PSMA-617

## Abstract

With the increasing importance of nuclear medicine therapy in overall cancer care and the introduction of ^177^Lu-PSMA-617 to Japan being imminent, the Policy Recommendations for Promoting Nuclear Medicine Therapy in Japan from the Working Group for Promoting Nuclear Medicine Therapy of the Japan Society of Clinical Oncology (JSCO) aim to identify issues related to nuclear medicine therapy and propose measures to address them, in order to further advance nuclear medicine therapy in Japan. It is necessary to create an environment in which each medical institution can proactively introduce nuclear medicine therapy by taking measures that target discharge criteria, medical fees, and human resource development and link them organically. The Working Group believes that it is necessary to continue examining long-term issues taking into account the changes that have occurred since the introduction of ^177^Lu-PSMA-617, as well as trends in nuclear medicine therapy other than ^177^Lu-PSMA-617 that are expected to be introduced in the future. In addition, as the “Action Plans for Promoting the Production and Use of Medical Radioisotopes” have been formulated and the advancement of nuclear medicine therapy has become one of the nation’s major policy priorities, JSCO will continue to actively work with relevant ministries and local governments to resolve the various issues of nuclear medicine therapy, including the smooth introduction of ^177^Lu-PSMA-617.

## Foreword

**Fig. 1:**
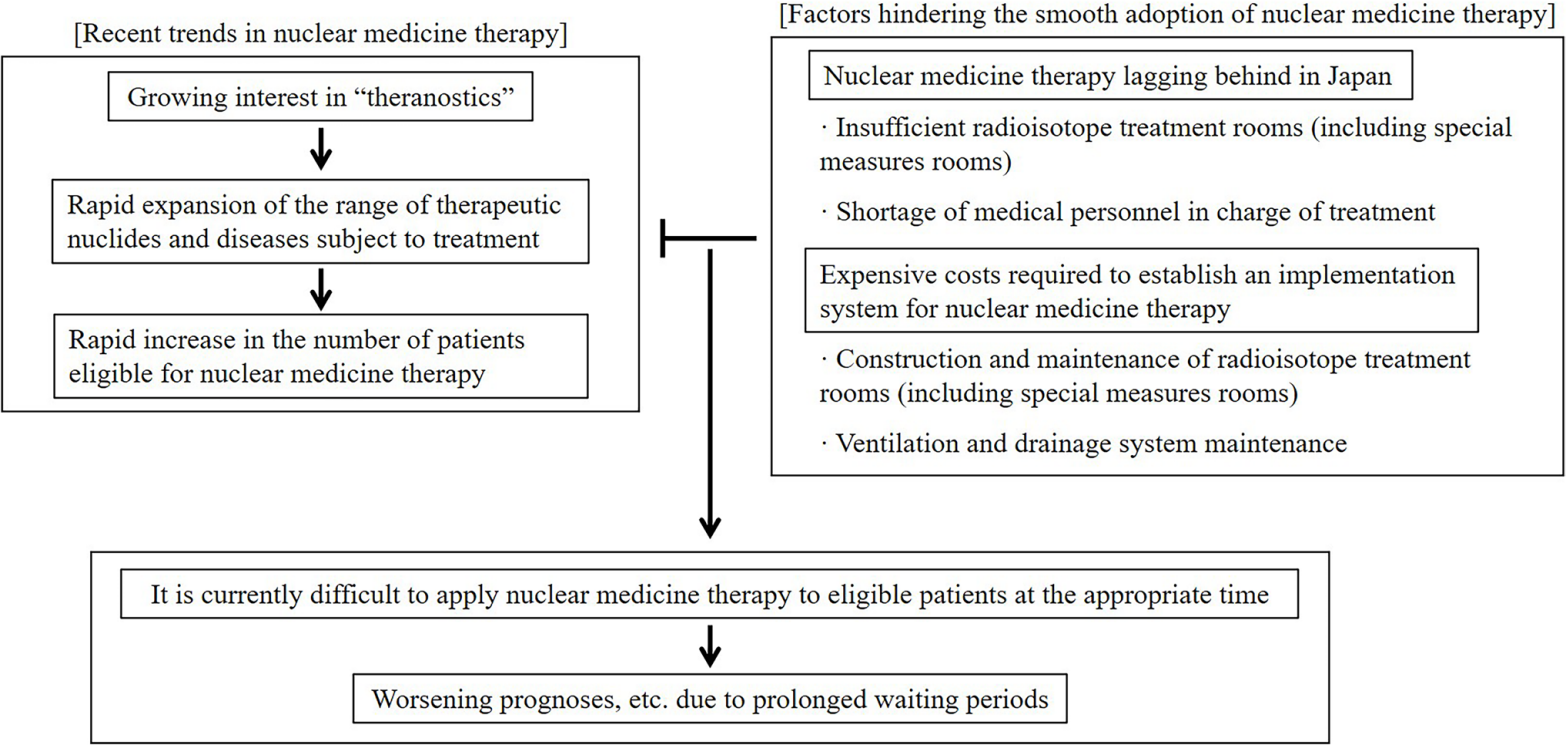
Current state of nuclear medicine therapy in Japan

Nuclear medicine therapy (radionuclide therapy) is a treatment in which a radionuclide, either bound to a substance that specifically accumulates in molecules expressed in the target tissue or used on its own, is selectively accumulated in a lesion and irradiated with radiation. Among these, the therapeutic concept of performing diagnostics and therapeutics in a continuous process by labeling substances that specifically bind to target molecules with diagnostic or therapeutic nuclides is often referred to as “theranostics.”[1] Nuclear medicine therapy is a treatment that has garnered a great deal of attention in recent years as a modality of cancer treatment. Along with the active development of new drugs, it is expected that the range of diseases that can be treated will expand rapidly in the future. However, in Japan, the expansion of facilities to perform nuclear medicine therapy is not easy due to complex regulations, with the spread of nuclear medicine therapy significantly delayed compared to Western countries due to a number of issues such as a shortage of personnel involved in nuclear medicine therapy and an inadequate educational system (Fig. 1).

Under such circumstances, ^177^Lu-PSMA-617 for prostate cancer [2], which has already been approved overseas and is spreading worldwide, is currently undergoing review for approval in Japan. Regarding ^177^Lu-PSMA-617, the number of patients with metastatic castration-resistant prostate cancer, who are the targets of this treatment, is significantly higher than that of diseases historically targeted by nuclear medicine therapy, leading us to believe that the introduction of this treatment will mark a major turning point for nuclear medicine therapy in Japan. That said, it is expected that the problems associated with nuclear medicine therapy as described above will become more apparent, creating a significant discrepancy between the number of patients requiring ^177^Lu-PSMA-617 and the number of times this therapy can be performed. In light of the above, it is an urgent issue for medical professionals involved in nuclear medicine therapy to once again clarify the current issues related to nuclear medicine therapy and examine solutions thereto in order to rapidly develop an implementation system for nuclear medicine therapy in Japan.

These recommendations summarize the issues related to nuclear medicine therapy in Japan and potential future issues identified by the Working Group for Promoting Nuclear Medicine Therapy of the Japan Society of Clinical Oncology, and propose concrete solutions to these issues. These recommendations are primarily intended for medical professionals. Moreover, it should be understood that much of the content mentioned in these evidence-based guidelines are unfamiliar, so these recommendations are based on expert consensus. Although these recommendations were compiled just prior to the introduction of ^177^Lu-PSMA-617, multiple nuclear medicine therapies targeting PSMA are currently being developed in addition to ^177^Lu-PSMA-617. The Working Group for Promoting Nuclear Medicine Therapy of the Japan Society of Clinical Oncology believes that it is necessary to continue examining long-term issues taking into account the changes that have occurred since the introduction of ^177^Lu-PSMA-617, as well as trends in nuclear medicine therapy other than radiolabeled PSMA ligands represented by ^177^Lu-PSMA-617 that are expected to be introduced in the future.

## Current state of nuclear medicine therapy in Japan

1. Action Plans for Promoting the Production and Use of Medical Radioisotopes.
  The “Action Plans for Promoting the Production and Use of Medical Radioisotopes” (https://www.aec.go.jp/kettei/kettei/20220531.pdf) were formulated by the Cabinet Office Atomic Energy Commission in 2022. These plans were developed in response to growing expectations for nuclear medicine therapy and global interest in “theranostics.” The aim of these plans is to contribute to public welfare and ensure international advantages from the perspective of economic security by realizing the mass production of domestic radioisotopes and enhancing our medical system through the widespread adoption of diagnosis and treatment using radioisotopes (Fig. 2). Within these plans, the following four items are listed as goals to be achieved in the next 10 years.

**Fig. 2:**
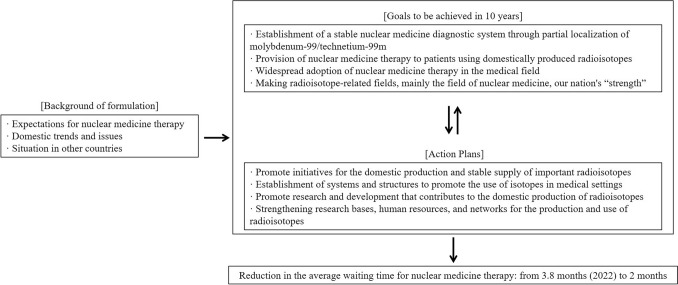
Outline of the action plans for promoting the production and use of medical radioisotopes
  The action plans to achieve these goals are summarized in accordance with the following items, with these plans also specified in important government policies such as the Fourth Basic Plan to Promote Cancer Control Programs (Cabinet Decision of March 28, 2023) (https://www.mhlw.go.jp/content/10901000/001091843.pdf).
  As these action plans progress and as the development of ^177^Lu-PSMA-617 and many other nuclear medicine therapies targeting PSMA advances and nuclear medicine therapies enter a new phase, these recommendations examine issues related to nuclear medicine therapy in Japan along with the countermeasures thereto, focusing particularly on the items of "establishment of systems and structures to promote the use of isotopes in medical settings" and "strengthening research bases, human resources, and networks for the production and use of radioisotopes," from the perspective of medical professionals in the Japan Society of Clinical Oncology, while taking an overview of nuclear medicine therapies including this therapy.
2. Nuclear medicine therapies approved in Japan and ^177^Lu-PSMA-617.
  Typical nuclear medicine therapies approved in Japan include radioactive iodine (^131^I) therapy for thyroid cancer and hyperthyroidism [3]. In addition, there are nuclear medicine therapies for castration-resistant prostate cancer with bone metastases and relapsed/refractory malignant lymphoma. Further, in 2021, ^131^I-MIBG therapy [4] for malignant pheochromocytoma/paraganglioma and ^177^Lu-DOTATATE therapy [5] for neuroendocrine tumors were approved as new nuclear medicine therapies and are becoming increasingly more common.
  Although these recommendations cover nuclear medicine therapy in general, an overview of ^177^Lu-PSMA-617 in particular will be provided herein, considering the current state in which a sense of crisis over the availability of nuclear medicine therapy systems is growing in the run-up to the introduction of this therapy. In 2021, the usefulness of ^177^Lu-PSMA-617 for metastatic castration-resistant prostate cancer was revealed based on the results of a large phase III clinical study (the VISION study) [2]. This study demonstrated that a combination of ^177^Lu-PSMA-617 and standard therapy effectively extended overall survival and progression-free survival compared to standard therapy alone, in addition to reducing pain caused by bone metastases and improving patients’ quality of life. In addition, for diagnosis, the treatment is performed after confirming PSMA expression in the target lesion in advance using ^68^ Ga-PSMA PET/CT, so it is also a revolutionary treatment in that it can maximize the therapeutic effect while avoiding unnecessary treatment. ^177^Lu-PSMA-617 is being increasingly used in actual clinical settings overseas, with an application for approval having already been made and currently under review in Japan. According to the “Investigation Report on the Medical Environment Surrounding Nuclear Medicine Therapy” (https://www.mizuho-rt.co.jp/archive/topics/pdf/2024_rnt01.pdf) by the investigation and study group on the environment surrounding nuclear medicine therapy, while the number of patients hospitalized for radioactive iodine therapy for thyroid cancer in the past five years was approximately 3,000/year, the number of patients eligible for nuclear medicine therapy with ^177^Lu-PSMA-617 in five years is expected to reach approximately 7,000 to 26,000, taking into consideration the multiple pathophysiologies for which ^177^Lu-PSMA-617 may be adopted and the degree of independence of patients from the perspective of radiation safety management. Regarding metastatic castration-resistant prostate cancer, there are currently multiple standard treatments available. Although ^177^Lu-PSMA-617 is not necessarily selected for all the patients described above, it is certain that the demand for nuclear medicine therapy for prostate cancer patients will significantly increase given that the indications for this therapy are expected to expand beyond metastatic castration-resistant prostate cancer and that other nuclear medicine therapies for advanced prostate cancer are expected to be introduced [6, 7].

## Issues related to nuclear medicine therapy in Japan

1. Shortage of radioisotope treatment rooms.
  According to the “9th National Nuclear Medicine Practice Survey Report” (https://www.jrias.or.jp/pdf/9th_kakuigakujitaityousa_2023_72_1_49.pdf) issued by the Japan Radioisotope Association, the number of beds in radiation treatment rooms (excluding special measures rooms) in operation in 2022 was 160 (66 facilities). Most of these are used for radioactive iodine therapy for thyroid cancer; however, the average waiting time has already reached 111.5 days according to the “Survey Report on the Operation Status of RI Treatment Rooms for Radioactive Iodine Therapy for Thyroid Cancer (2022)” (https://www.jstage.jst.go.jp/article/kakuigaku/59/1/59_rp.2240/_pdf/-char/en). On the other hand, the Action Plans for Promoting the Production and Use of Medical Radioisotopes set a goal of “reducing the average waiting period until the administration of nuclear medicine therapy, including new nuclear medicine therapy drugs that will be introduced in the future, to an average of two months by FY2030.” According to current estimates, the number of beds required to treat the number of patients indicated for radioactive iodine therapy is estimated to be 133. Assuming that the average annual patient capacity per bed in the radiation treatment room is 27.4 (including thyroid cancer, hyperthyroidism, malignant pheochromocytoma/paraganglioma), if radiation therapy rooms are used efficiently, there will not necessarily be an acute shortage of such rooms. However, when ^177^Lu-PSMA-617 is introduced in the future, it is clear that there will be a severe shortage of beds required for nuclear medicine therapy due to the large number of patients indicated for the treatment. In the “Investigation Report on the Medical Environment Surrounding Nuclear Medicine Therapy” (https://www.mizuho-rt.co.jp/archive/topics/pdf/2024_rnt01.pdf), based on an analysis taking into consideration the predicted number of indicated patients and the difference in bed management (the number of patients treated per week), it is estimated that the number of beds required for nuclear medicine therapy for prostate cancer and neuroendocrine tumors using ^177^Lu as of 2033 will be between 470 and 3,314.
  In an analysis based on a simulation model that took into account the future increased demand for nuclear medicine therapy, reported by Mizowaki et al., assuming that 25% of patients with advanced PSMA-positive metastatic castration-resistant prostate cancer will choose this treatment, it was predicted that the total number of patients eligible for nuclear medicine therapy would increase by approximately 1.5- to 1.8-fold as of 2022 (approximately 6,500 people/year to approximately 10,000 to 11,500 people/year)[8]. In addition, it was suggested that under the current treatment system, there may be a sharp increase in long-term waiting patients with a waiting period of more than 6 months for nuclear medicine therapy following the introduction of ^177^Lu-PSMA-617. Specifically, it was anticipated that after four years following the introduction of ^177^Lu-PSMA-617, the cumulative number of patients waiting for more than 180 days to treat prostate and thyroid cancers and more than 365 days to treat other diseases, including neuroendocrine tumors, will increase approximately fourfold. Furthermore, there have been occasional reports of patients adversely affected due to such delayed medical intervention. In the United States, where ^177^Lu-PSMA-617 was introduced prior to Japan, it has been reported that 6 of the 127 patients (5%) who agreed to the treatment died before receiving treatment due to the prolonged waiting period (41 days as of May 2022 to 96 days as of October 2022) [9].
2. Insufficient maintenance of radiation protection equipment.
  Each country’s regulations on radiation protection are established based on the recommendations of international organizations such as the International Commission on Radiological Protection (ICRP) and the International Atomic Energy Agency (IAEA). In Japan, details are stipulated in medical care act, regulations of the implementation thereof, and related notifications. Regarding the new construction and renovation of radioisotope treatment rooms, from the perspective of radiation protection, it is necessary to provide shielding structures, equipment near interior walls, floors, and entrances, and structural equipment such as drainage and air conditioning systems, as well as drainage and exhaust equipment designed to reduce the concentration of radioisotopes in drainage and exhaust air to below a certain concentration (concentration limit). The aforementioned simulation by Mizowaki et al. has indicated that, depending on the facility, not only will there be a shortage of treatment beds, but a lack of drainage capacity may also lead to extended waiting periods; however, because such radiation protection equipment is expensive, promoting the installation thereof is not necessarily easy. Further, simulations have demonstrated that even with an increase in the number of rooms that can administer nuclear medicine therapy, it may not be possible to provide nuclear medicine therapy efficiently if the development of radiation protection facilities, such as drainage capacity, is insufficient [8].
3. Shortage of personnel involved in nuclear medicine therapy.
  As has been pointed out, there is a shortage of personnel related to nuclear medicine therapy, with currently only approximately 6000 diagnostic radiologists (https://www.radiology.jp), approximately 1,400 radiation oncologists (https://www.jastro.or.jp), and approximately 1,500 nuclear medicine specialists (https://jsnm.org). In addition, nuclear medicine exists as a subfield of diagnostic radiology in many hospitals, with only a limited number of facilities having nuclear medicine as an independent department. For this reason, there are many facilities that only conduct imaging tests among medical care related to nuclear medicine, with quite a few facilities where doctors specializing in radiotherapy or diagnostic radiology are in charge of nuclear medicine therapy. According to the “Survey Report on the Operation Status of RI Treatment Rooms for Radioactive Iodine Therapy for Thyroid Cancer (2022)” (https://www.jstage.jst.go.jp/article/kakuigaku/59/1/59_rp.2240/_pdf/-char/ja) conducted in 2021 among medical facilities that have radiation treatment rooms, among 56 responding facilities, 35 facilities (63%) had nuclear medicine specialists performing nuclear medicine therapy, while 33 facilities (59%) had radiation oncologists performing nuclear medicine therapy, with 13 of these 33 facilities (39%) having no nuclear medicine specialists. Moreover, it has been pointed out that there is also a shortage of non-physician specialists required in nuclear medicine departments (including radiologic technologists, nurses, pharmacists, etc.). In Europe and the United States, specialists such as nuclear medicine technologists, radiopharmacists, radiation safety specialists, and medical physicists are assigned to these facilities (https://www.healthpolicypartnership.com/app/uploads/Health-system-readiness-for-radioligand-therapy-in-the-US-situation-analysis-report.pdf); however, in Japan, the differentiation of these professions has not progressed, and although the certification of nuclear medicine technologists, nuclear medicine clinical nurses, and nuclear medicine certified pharmacists has been introduced, the number of certified personnel at the time of writing these recommendations was only 687, 292, and 59, respectively, indicating insufficient adoption of these qualifications (https://jsnm.org).

## Recommendations for solving issues related to nuclear medicine therapy in Japan

1. Effective use of special measures rooms.
  Among the hospital rooms that admit patients undergoing nuclear medicine therapy, special measures rooms refer to general rooms, etc., that are prescribed in the Article 30-12, paragraph 2 of the Enforcement Regulations on the Medical Care Act, and have taken the same measures as the radiation treatment rooms prescribed in Paragraph 1 of said article. Upon conducting a clinical study of ^177^Lu-DOTATATE therapy for somatostatin receptor-positive neuroendocrine tumors since 2017, given the current shortage of radiation treatment rooms and the extremely small amount of radiation released into the air after ^177^Lu administration, patients were admitted to special measures rooms for the first time. The use of these special measures rooms was subject to an exception to Article 30–15 of the Enforcement Regulations of the Medical Care Act, which requires patients who have been administered radiopharmaceuticals be admitted to the radiation treatment rooms, and which allows patients to be admitted to a room other than a radiation treatment room if "appropriate protective measures and contamination prevention measures are taken." In addition, the specific details of appropriate protective measures and anti-pollution measures were in accordance with the “Manual on Standards, Management, and Operation, and Code of Conduct concerning Special Measures Rooms” prepared by the relevant academic societies based on a study from Health and Labour Sciences Research, and the special measures rooms were used in accordance therewith. Subsequently, with the pharmaceutical approval of ^177^Lu-DOTATATE therapy, the notification “Regarding the Discharge of Patients Who Received Radiopharmaceuticals” (https://www.mhlw.go.jp/content/10800000/001063188.pdf) by the Director of the Medical Care Planning Division, Health Policy Bureau, Ministry of Health, Labour and Welfare also allowed patients who received ^177^Lu-DOTATATE to be admitted to the special measures rooms, a point which was clarified in the amendment to the Enforcement Regulations of the Medical Care Act in 2022 by adding special measures rooms to the structural equipment standards through the new establishment of Article 30-12, Paragraph 2 of the Enforcement Regulations of the Medical Care Act. These special measures rooms are advantageous in that they can be constructed inexpensively, without requiring the high costs (tens of millions of yen to hundreds of millions of yen) required to establish and maintain the aforementioned radiation treatment rooms. The utilization of special measures rooms has been promoted in the “Action Plans for Promoting the Production and Use of Medical Radioisotopes” as a way of increasing the number of beds available for nuclear medicine therapy. Regarding ^177^Lu-PSMA-617, the “Manual for the Appropriate Use of Nuclear Medicine Therapy using Lutetium-177-Labeled PSMA-Specific Ligand (Lu-177-PSMA-617) (2nd edition)” (https://jsnm.org/archives/8096/) approved by the Japanese Society of Nuclear Medicine allows patients administered ^177^Lu-PSMA-617 to be admitted to the special measures rooms, so taking into consideration the fact that ^177^Lu-PSMA-617 is currently under review for approval, it is necessary to increase the number of special measures rooms in order to prepare for the implementation of this therapy. Specifically, it is hoped that the establishment of special measures rooms will be promoted in the 461 designated cancer hospitals as of April 1, 2024 or equivalent core institutions existing nationwide. In order to achieve this, during the interim review of the eighth medical plan (planning period 2024–2029) (https://www.mhlw.go.jp/content/10800000/001106486.pdf) scheduled for FY2026, we believe that the “Guidelines for Establishing a Medical System for Diseases, Services, and Home-Based Care” presented by the government should include a description of the nuclear medicine therapy functions (bases) and that prefectural plans should aim to define specific medical facility functions based thereon. Moreover, when actually operating these special measures rooms, it is important to pay attention to reasonable shielding suited to the circumstances of each facility and take appropriate protective measures taking into account an evaluation of the risk of contamination, etc. It is hoped that examples related to specific methods will be compiled and shared with governmental bodies and relevant academic societies.
2. Efficient management of the quantity of radionuclide used.
  It is clear that efficiently managing the quantity of nuclides used will lead to the smooth provision of nuclear medicine therapy. Specifically, each facility should consider reviewing the following matters: reviewing and organizing the nuclides to be used; exhausting the air via 24-h air conditioning; diluting wastewater up to tenfold; introducing multi-channel analyzers for radiation concentration management in wastewater; and using the effective half-life of nuclides and the occupancy factor of adjacent patients when calculating shielding for special measures rooms.
  However, it is not easy to put these measures into practice, as they require not only advanced expertise but also very expensive renovations to hospital facilities such as the addition of water storage tanks, the installation of septic tanks, etc., and the purchase of other items. In addition to revising medical fees related to nuclear medicine therapy, which will be described later, it should be emphasized that we should also proactively take advantage of subsidies such as the Regional Integrated Medical and Nursing Care Fund and lobby the government to further improve the treatment environment.
3. Improving the management of discharge criteria.
  In order to achieve the expansion of treatment capacity, we believe that aiming to increase the number of beds and the number of treatments per bed is an effective measure. One of the factors affecting the occupancy rate of beds used for nuclear medicine therapy is the discharge criteria. From the perspective of preventing unnecessary exposure of the public and caregivers, for each nuclide, the criteria based on the dosage and the amount of residual radiation in the body, the criteria based on the measured dose equivalent rate, and the criteria based on the cumulative dose calculation per patient are defined in order to meet the dose limit of the general public (1 mSv/year) and the dose limit of caregivers (5 mSv/treatment) (Table 1). If the discharge criteria could be relaxed, it would lead to an increase in bed occupancy and would likely be effective in expanding treatment capacity; however, this will not be addressed here. Therefore, these recommendations encourage discharging patients after properly evaluating the condition of individual patients based on cumulative dose calculations for each patient. For example, in radioactive iodine therapy, patients may be discharged if either the criteria based on the dosage or amount of residual radiation in the body (500 MBq or less) or the criteria based on the measured dose rate (30 μSv/h or less, actual measurement is required) is met. However, in the case of ablation treatment using ^131^I, according to “Outpatient Treatment Using ^131^I (1,110 MBq) for Thyroid Remnant Ablation” prepared by the Japanese Society of Nuclear Medicine and related societies, the dosage is already 1,110 MBq to start with, which is higher than the discharge criteria (500 MBq or less). Nevertheless, even if the dose rate based on the “criteria based on the measured dose rate” exceeds 30 μSv/h, patients can be discharged on the same day by reducing the accumulated dose of caregivers and the public to 5 mSv/treatment, 1 mSv/year or less, respectively, by imposing certain conditions such as having the patient and the family follow the guidance for patients and their families when returning home after outpatient treatment under the guidance and supervision of a nuclear medicine specialist. Regarding ^223^Ra and ^177^Lu, in accordance with the respective implementation guidelines, patients can also be discharged in accordance with the “criteria based on the cumulative dose calculations for each patient.” Since it is essential for the patient and family to properly manage this “criteria based on cumulative dose calculations per patient,” careful judgment should be made based on patient-specific ADLs, cognitive function, etc. That said, at the time of discharge following administration, if the patient and their family (or caregivers) have received a thorough explanation regarding this therapy in advance by a specialist and it is determined that they are able to follow the precautions in their daily lives, then proactively utilizing this discharge criteria is believed likely to shorten the hospitalization period and further improve the occupancy rate of hospital beds. Currently, the only implementation guidelines that can be clinically applied as discharge criteria for ^177^Lu are the “Manual for Appropriate Use of Nuclear Medicine Therapy using Lutetium Oxodotreotide (^177^Lu) Injection” (https://www.jrias.or.jp/pdf/Lu-177manual_v1.pdf) and the "Manual for Appropriate Use of Nuclear Medicine Therapy using Lutetium-177-Labeled PSMA-Specific Ligand (Lu-177-PSMA-617) (2nd edition)”; therefore, it is considered necessary to revise or formulate new implementation guidelines in accordance with ^177^Lu-PSMA-617 going forward.

**Table 1:** Summary of discharge criteria from “Discharge of Patients Who Received Radiopharmaceuticals”

(a) Discharge criteria based on dosage		
Radionuclide	Dosage or amount of residual radiation in the body	
^131^I	500 MBq	
^89^Sr	200 MBq	
^90^Y	1184 MBq	

b) Discharge criteria based on measured dose		
Radionuclide	1 cm dose equivalent rate at 1 m from the patient’s body surface	
^131^I	30 µSv/h	

c) Discharge criteria based on cumulative dose calculations per patient		
Radionuclide	Indication	Conditions
^131^I	Thyroid remnant ablation treatment following total thyroidectomy for differentiated thyroid carcinoma without distant metastasis	Conduct in accordance with “Outpatient Treatment Using ^131^I (1,110 MBq) for Thyroid Remnant Ablation”
^223^Ra	Treatment for castration-resistant prostate cancer with bone metastases	Conduct in accordance with the “Manual for Appropriate Use of Internal Therapy Using Radium Chloride (^223^Ra) Injection” Administer 55 kBq/kg up to 6 doses in 4-week intervals
^177^Lu	Treatment for somatostatin receptor-positive neuroendocrine tumors	Conduct in accordance with the “Manual for the Appropriate Use of Nuclear Medicine Treatment Using Lutetium Oxodotreotide (^177^Lu) Injection” Administer 7.4 GBq/dose up to 4 doses in 8-week intervals
4. Efforts to revise medical fees.
  When a patient is admitted to a radiation treatment room, in addition to the basic hospitalization fee, the “Radiation Treatment Room Management Surcharge” can be calculated as part of the basic hospitalization fee surcharge. In the 2022 revision of medical fees, the points for the “Radiation Treatment Room Management Surcharge” for treatment using therapeutic radioisotopes were significantly increased (from 25,000 yen points per day to 63,700 yen points per day). In addition to conventional radiation treatment rooms, it is now possible to also calculate the “Radiation Treatment Room Management Surcharge” for special measures rooms if they meet certain criteria. However, as mentioned above, the construction of radiation treatment rooms and radiation protection equipment is extremely expensive, and even for special measures rooms, when considering maintenance costs, labor costs of relevant staff, and costs required for the provision of education, radiation exposure management, legal procedures, etc., the medical fees for nuclear medicine therapy are still insufficient and could potentially become a serious concern for medical institutions planning to set up new beds. In fact, in its request for revision of medical fees in 2022, the number of points required per bed per day as calculated by the Japanese Society of Nuclear Medicine based on the maintenance costs pertaining to the radiation treatment room was 95,330 yen. The working group also believes that we should aim to further increase the aforementioned Radiation Treatment Room Management Surcharge. For example, while giving due and careful consideration to the increase in public exposure, a new facility standard stating “thorough education and guidance can be provided to the patient at the time of discharge” should be established to achieve a further increase in the Radiation Treatment Room Management Surcharge in facilities that meet the standard. A measure such as the proactive use of “criteria based on cumulative dose calculations for each patient” is believed to be worth considering as it would be expected to contribute to increasing the number of treatable patients by making more efficient use of limited hospital rooms. In addition, with regard to the radioisotope-based internal therapy management surcharge stipulated for each disease, in light of the current situation that the smooth introduction of ^177^Lu-PSMA-617 in particular will require significant expenses, including the operation of ^68^ Ga-PSMA PET/CT, which is essential for diagnosis, we should consider advocating to obtain the highest possible surcharge.
5. Human resource development.
  In order to further advance nuclear medicine therapy in Japan and ensure sufficient treatment opportunities, in addition to expanding the number of hospital beds, resolving the shortage of personnel involved in nuclear medicine therapy is also a very important issue. The aforementioned action plans also include plans to advance considerations related to how education on radiation and radioisotopes is valued, as well as how physicians and medical-related occupations engaged in nuclear medicine therapy and radiation treatment, including nuclear medicine specialists, radiologic technologists, and certified medical physicists, can fully demonstrate their expertise. We, as a working group, believe it is important to encourage employees to actively attend both training on the safe handling of pharmaceuticals and various seminars organized by radiological academic societies and the Japan Radioisotope Association for their respective professions, in order to acquire fundamental knowledge and skills. In fact, it is normally impossible to organize a nursing unit consisting of only radiation treatment rooms or special measures rooms, and the nurses have to be in charge managing both patients to be treated for nuclear medicine and patients in the main department. Therefore, their knowledge of nuclear medicine therapy is often insufficient, which could potentially become an obstacle to the smooth implementation of nuclear medicine therapy. For this reason, we emphasize the importance of improving the educational system for nuclear medicine therapy, especially for nurses. Furthermore, the Japan Society of Clinical Oncology is seeking to obtain funding from companies, etc., for the sole purpose of educational projects, in addition to competitive funding in order to enhance educational opportunities focusing on nuclear medicine therapy. Moreover, it should be emphasized that at facilities which implement nuclear medicine therapy, providing opportunities for periodical information sharing between multiple occupations within hospitals and continuously reviewing and improving the implementation system for nuclear medicine therapy will also contribute to human resource development.

## Conclusion

With the increasing importance of nuclear medicine therapy in overall cancer care and the introduction of ^177^Lu-PSMA-617 being imminent, these recommendations aim to identify issues related to nuclear medicine therapy and propose measures to address them, in order to further advance nuclear medicine therapy in Japan. First, while expanding treatment capacity is an urgent issue, it is also necessary to increase the number of treatment beds and improve radiation protection equipment related to ventilation, drainage, shielding, etc. Furthermore, as it is expected that operating under a limited budget and equipment will be necessary for the time being, there will be a need to devise ways to ensure efficient operation and management. We believe it is possible to effectively use the limited number of beds based on the differences in the characteristics of each nuclide, taking into account the preferential use of existing radiation treatment rooms for treatment using ^131^I and the significant increase in the number of eligible patients for treatment using ^177^Lu in the future, by establishing and promoting the use of special measures rooms that are relatively inexpensive to build and actively utilizing the “criteria based on the cumulative dose calculations for each patient” for discharge criteria. There is also an urgent need to resolve the shortage of personnel involved in nuclear medicine therapy. In Japan, because there is no subdivision of occupations related to nuclear medicine therapy, it is necessary to actively hold trainings, education seminars, etc., for existing medical professionals in order to develop human resources with sufficient expertise.

**Fig. 3:**
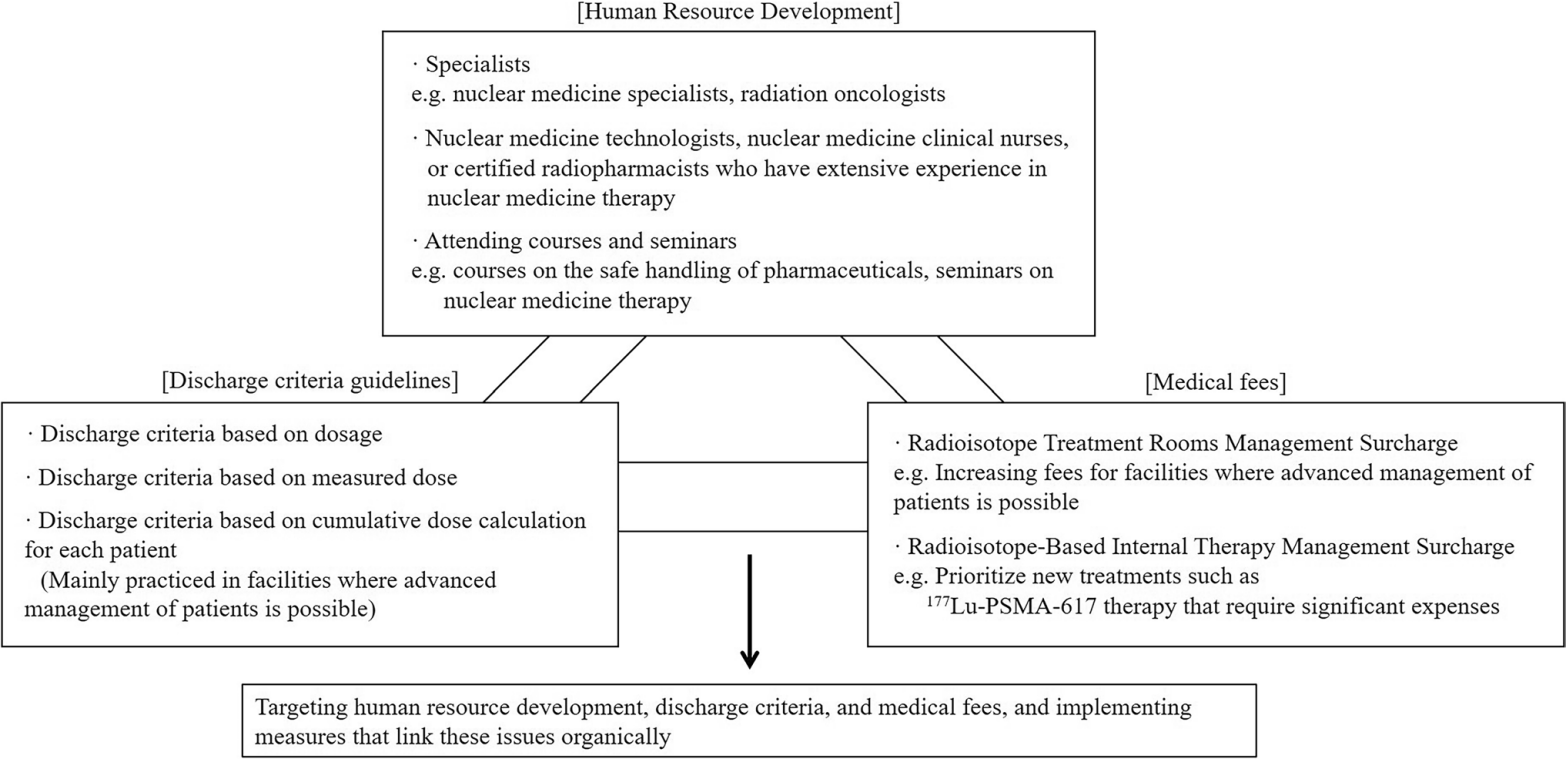
Initiatives required to promote nuclear medicine therapy in Japan

However, because the expenses for establishing such an implementation system for nuclear medicine therapy are so high, in order to reduce the burden on medical institutions, we should aim to increase the Radiation Treatment Room Management Surcharge for facilities that are capable of providing advanced education and guidance to patients to operate the aforementioned “criteria based on cumulative dose calculation for each patient” and increase the radioisotope-based internal therapy management surcharge for new treatments such as ^177^Lu-PSMA-617 that have a large number of eligible patients. With this in mind, it is necessary to create an environment in which each medical institution can proactively introduce nuclear medicine therapy by taking measures that target discharge criteria, medical fees, and human resource development and link them organically (Fig. 3).

The “Action Plans for Promoting the Production and Use of Medical Radioisotopes” have been formulated and the advancement of nuclear medicine therapy has become one of the nation’s major policy priorities. In fact, in addition to ^177^Lu-PSMA-617, many promising nuclear medicine therapy drugs are currently under development. The Japan Society of Clinical Oncology will continue to actively work with relevant ministries and local governments to resolve the various issues of nuclear medicine therapy, including the smooth introduction of ^177^Lu-PSMA-617.
