# Correction to: Policy recommendations for promoting nuclear medicine therapy in Japan 2025, from the Working Group for promoting nuclear medicine therapy of the Japan Society of Clinical Oncology

**DOI:** 10.1007/s10147-025-02925-9

**Published:** 2026-01-08

**Authors:** Kotaro Suzuki, Hideaki Miyake, Anri Inaki, Shoko Takano, Yasutoshi Kuboki, Takashi Mizowaki, Katsumasa Nakamura, Makoto Ueno, Shigemi Matsumoto, Daisuke Obinata, Tohru Nakagawa, Masato Murakami, Yoshiyuki Majima, Megumu Yokono, Masao Namba, Takayuki Yoshino

**Affiliations:** 1 Division of Urology, Kobe University Graduate School of Medicine, Kobe, Japan; 2 Division of Functional Imaging, Exploratory Oncology Research and Clinical Trial Center, National Cancer Center, Kashiwa, Japan; 3 Department of Nuclear Medicine, Yokohama City University Hospital, Yokohama, Japan; 4 Department of Experimental Therapeutics, National Cancer Center Hospital East, Kashiwa, Japan; 5 Department of Radiation Oncology and Image-Applied Therapy, Graduate School of Medicine, Kyoto University, Kyoto, Japan; 6 Department of Radiation Oncology, Hamamatsu University School of Medicine, Hamamatsu, Japan; 7 Department of Gastroenterology, Kanagawa Cancer Center, Yokohama, Japan; 8 Department of Real World Data R and D, Graduate School of Medicine, Kyoto University, Kyoto, Japan; 9 Department of Urology, Nihon University School of Medicine, Tokyo, Japan; 10 Department of Urology, Teikyo University School of Medicine, Tokyo, Japan; 11 Japan Radiopharmaceuticals Association, Tokyo, Japan; 12 PanCAN Japan, Tokyo, Japan; 13 School of Social Sciences, Waseda University, Tokyo, Japan; 14 Planning Section of Radiopharmaceutical Division, Japan Radioisotope Association, Tokyo, Japan; 15 Department of Global Oncology, National Cancer Center Hospital East, Kashiwa, Japan

**Correction to: International Journal of Clinical Oncology** 10.1007/s10147-025-02858-3

The article Policy recommendations for promoting nuclear medicine therapy in Japan 2025, from the Working Group for promoting nuclear medicine therapy of the Japan Society of Clinical Oncology by Kotaro Suzuki, Hideaki Miyake, Anri Inaki, Shoko Takano, Yasutoshi Kuboki, Takashi Mizowaki, Katsumasa Nakamura, Makoto Ueno, Shigemi Matsumoto, Daisuke Obinata, Tohru Nakagawa, Masato Murakami, Yoshiyuki Majima, Megumu Yokono, Masao Namba, Takayuki Yoshino was originally published electronically on the publisher’s internet portal 10.1007/s10147-025-02858-3 on 30 September 2025 without Open Access.

With the author(s)’ decision to opt for Open Choice, the copyright of the article changed on November 20, 2025 © to The Author(s) 2025 and the article is forthwith distributed under the terms of the Creative Commons Attribution 4.0 International License (https://creativecommons.org/licenses/by/4.0/), which permits use, duplication, adaptation, distribution and reproduction in any medium or format, as long as you give appropriate credit to the original author(s) and the source, provide a link to the Creative Commons license, and indicate if changes were made.

The original article has been corrected.

